# Adsorbed States of Hydrogen on Platinum: A New Perspective

**DOI:** 10.1002/chem.201900351

**Published:** 2019-04-17

**Authors:** Stewart F. Parker, Sanghamitra Mukhopadhyay, Mónica Jiménez‐Ruiz, Peter W. Albers

**Affiliations:** ^1^ ISIS Facility STFC Rutherford Appleton Laboratory Chilton Didcot OX11 0QX UK; ^2^ Institut Laue-Langevin 71 avenue des Martyrs, CS 20156 38042 Grenoble, Cedex 9 France; ^3^ Evonik Technology & Infrastructure GmbH Rodenbacher Chaussee 4 63457 Hanau/Wolfgang Germany

**Keywords:** ab initio calculations, density functional calculations, neutron scattering, infrared spectroscopy, surface chemistry

## Abstract

The interaction of hydrogen with platinum is enormously important in many areas of catalysis. The most significant of these are in polymer electrolyte membrane fuel cells (PEMFC), in which carbon‐supported platinum is used to dissociate hydrogen gas at the anode. The nature of adsorbed hydrogen on platinum has been studied for many years on single‐crystal surfaces, on high‐surface area‐platinum metal (Raney platinum and platinum black), and on supported catalysts. Many forms of vibrational spectroscopy have played a key role in these studies, however, there is still no clear consensus as to the assignment of the spectra. In this work, ab initio molecular dynamics (AIMD) and lattice dynamics were used to study a 1.1 nm nanoparticle, Pt_44_H_80_. The results were compared to new inelastic neutron scattering spectra of hydrogen on platinum black and of a carbon‐supported platinum fuel cell catalyst and an assignment scheme that rationalises all previous data is proposed.

Platinum‐based catalysts are widely used throughout industry.^1^ Major applications include: the reduction of nitroarenes to aromatic aminoarenes for use in polyurethane manufacture,^2^ as a component in the three‐way automotive catalyst,^3^ as the anode in polymer electrolyte membrane fuel cells (PEMFC)^4^, and in chemotherapy.^5^ Many of the uses of platinum arise from the facile^6^ dissociation of dihydrogen at the catalyst surface. Thus, it is not surprising that the nature of adsorbed hydrogen on platinum surfaces has been studied for decades.^7^ Vibrational spectroscopy has played a key role in these studies and many forms of vibrational spectroscopy have been used to investigate adsorbed hydrogen on single‐crystal surfaces [in ultra‐high vacuum (UHV)^8^ and on electrodes^9^], on high‐surface‐area platinum metal^10^ and on supported catalysts.^11^ However, there is still no clear consensus as to the assignment of the spectra.

A recent computational study has shown^12^ that an initially ideal Pt_44_ octahedron, with only {111} facets, undergoes considerable reconstruction as hydrogen is added to produce a Pt_44_H_80_ *C*_2*h*_ tetradecahedron of *fcc* packing, with 8 {111} facets, 6 {100} facets and 18 apex Pt atoms (see Figure 1). This structure has 18 on‐top, 44 twofold, 18 threefold, 0 fourfold coordinated hydrogen and no subsurface hydrogen. The last observation is consistent with the extremely small solubility of hydrogen in platinum,^13^ in marked in contrast to the high hydrogen storage capability of palladium,^14^ irrespective of whether it is a metal black or supported nanoparticles.

**Figure 1:**
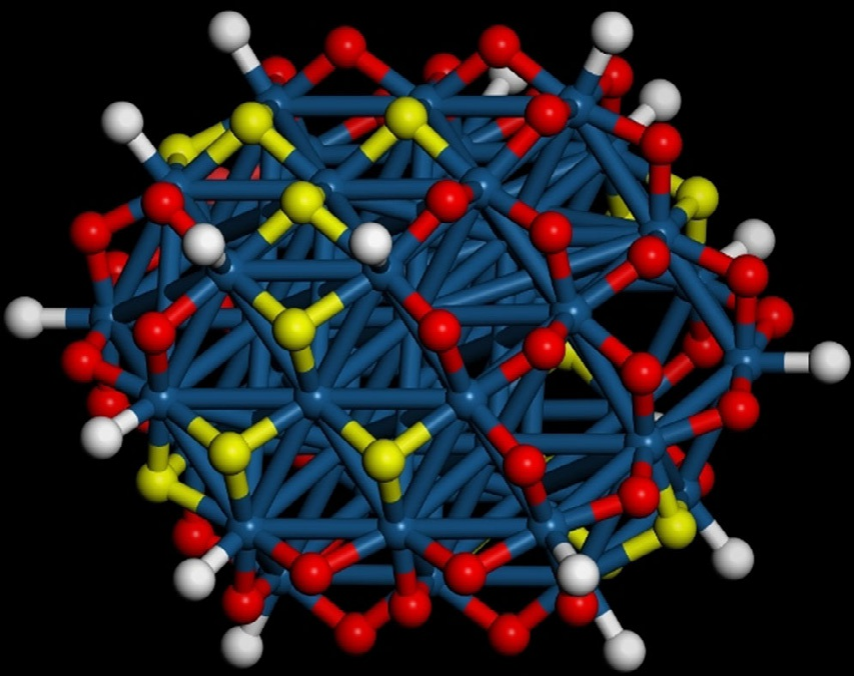
Structure of the 1.1 nm Pt_44_H_80_ *C*_2*h*_ tetradecahedron^12^ (Pt = dark blue, on‐top hydrogen = white, twofold hydrogen = red, threefold hydrogen = yellow).

In the present work, we have calculated the vibrational density of states (VDOS) of hydrogen on this reconstructed 1.1 nm platinum nanoparticle by both lattice dynamics (which uses the harmonic approximation) and by ab initio molecular dynamics (AIMD, which includes anharmonicity).

Figure 2 compares the inelastic neutron scattering (INS) spectrum of hydrogen on platinum black with those of the Pt_44_H_80_ nanoparticle calculated by lattice dynamics and the VDOS calculated by AIMD. The INS spectrum is used because INS is the only technique capable of observing all of the modes of the adsorbed species. This arises because there are no selection rules in INS spectroscopy; however, there is a bias, such that modes that involve displacement of ^1^H are those observed.^15^ (This is explained in more detail in the Supporting Information). INS spectroscopy is finding increasing use in studies of catalysts.^16^

**Figure 2:**
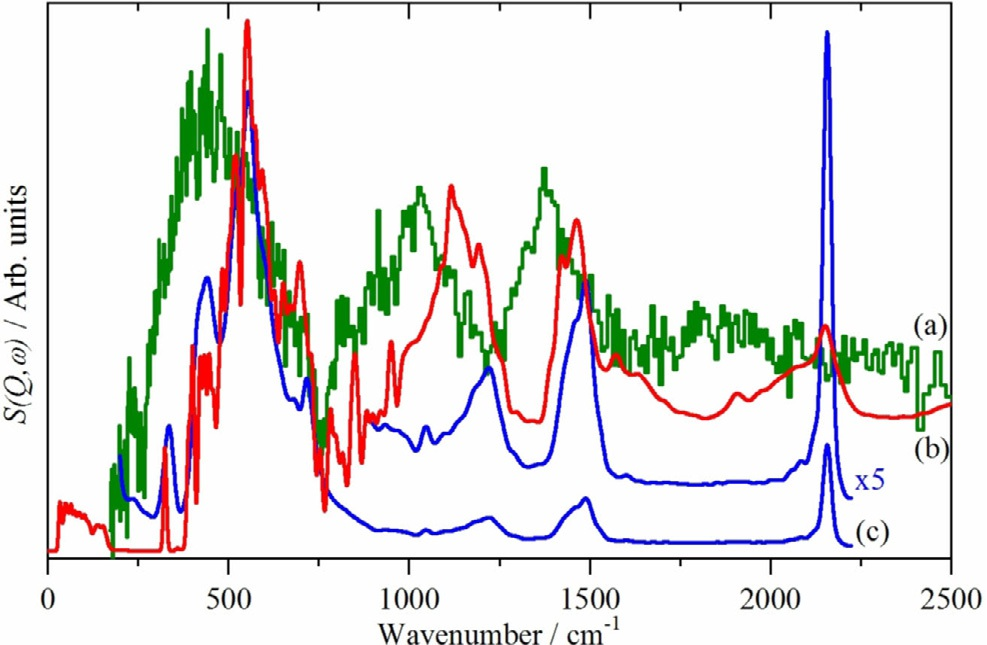
Comparison of: (a) the experimental INS spectrum of hydrogen on platinum black (olive) recorded on IN1‐Lagrange with that calculated by (b) lattice dynamics (red) and (c) AIMD (blue) for the Pt_44_H_80_ nanoparticle.

It is apparent that the lattice dynamics and AIMD calculations produce similar results. (The difference in the mode intensities between the lattice dynamics and the AIMD calculation is explained in the Supporting Information). Decomposing the spectra into the separate contributions from on‐top, twofold and threefold sites, Figure 3, shows why this is the case: both methods predict very similar transition energies. It has been suggested8e,8f that hydrogen in the threefold site experiences a significantly anharmonic potential, the similarity of the lattice dynamics (a harmonic calculation) and the AIMD (which includes anharmonicity) suggests this is not the case.

**Figure 3:**
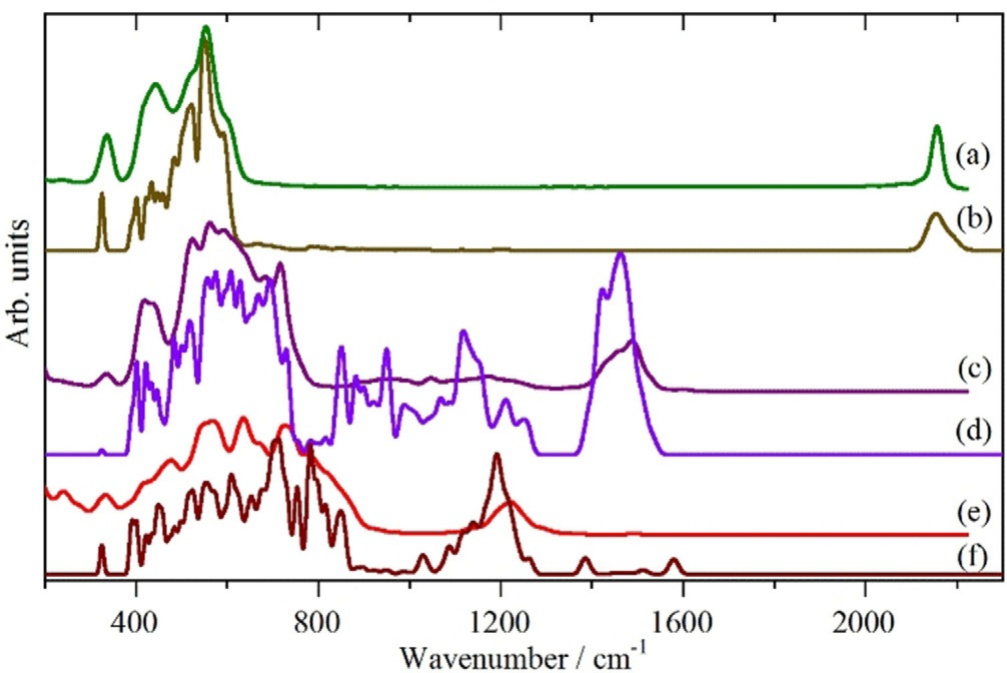
Contributions from the different sites to the total spectrum of the Pt_44_H_80_ nanoparticle: (a) and (b) on‐top, (c) and (d ) twofold, (e) and (f) threefold. (a), (c) and (e) contributions are from the AIMD calculation and (b), (d) and (f) are from the lattice dynamics calculation. Only the fundamental (0→1) transitions are shown.

We note that the calculation was for a 1.1 nm particle, whereas a fuel cell catalyst will have platinum particles in the of approximately 3±1 nm, because it has been shown^17^ that this is the optimum size for both the hydrogen oxidation reaction and the oxygen reduction reaction. The platinum black sample shown in Figure 2 consists of primary crystallites of varying size in the 3 to 10 nm range, which are grown together to form strongly bound large polydisperse aggregates and loosely co‐adherent agglomerates.^18^ However, the excellent agreement between the model system and the experimental data demonstrates that in both cases the spectra are dominated by the twofold bridge sites. Previous work11e has shown that the main peak at approximately 500 cm^−1^ narrows as the average particle size increases from 3 to 5 nm, but the overall profile is retained.

The relative contribution of the on‐top hydrogen will vary depending on the conditions, in particular it is only present with an overpressure of hydrogen. The Pt−H stretch mode has been observed by infrared11a,11b and INS spectroscopies^6^ on supported metal catalysts. Figure 4 shows infrared spectra of the on‐top Pt−H species on a variety of supports: a Pt (1 %)/Al_2_O_3_ hydrogenation catalyst, a Pt (10 %)/C fuel cell catalyst and the standard catalyst EuroPt1 [Pt (6 %)/SiO_2_].^19^ The associated bending mode has not been detected previously, because it is forbidden in the infrared spectrum by the metal surface selection rule. This is irrelevant for INS spectroscopy and Figure 5 shows the INS spectrum at the temperature of 15 K (at which H_2_ is a solid) of a Pt (58 %)/C catalyst with H_2_ present, Figure 5 a, and the same sample after briefly pumping at 77 K, which removes the H_2_ (and hence the on‐top hydrogen) but leaves the high coordination sites untouched, Figure 5 b. The features associated with solid H_2_ disappear and a mode at 480 cm^−1^ is attenuated, which is assigned to the Pt−H bending mode.

**Figure 4:**
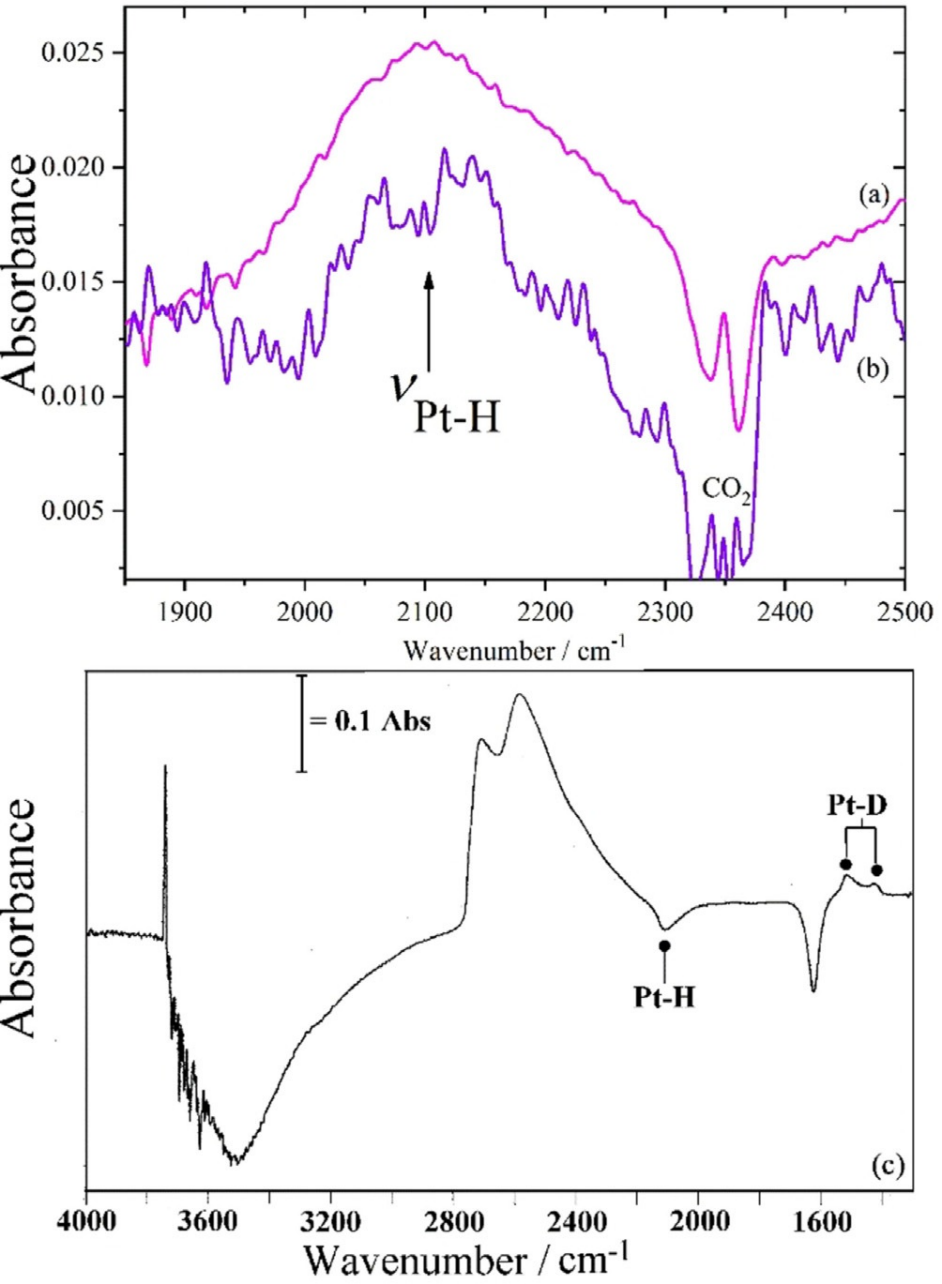
Diffuse reflectance infrared spectra of the Pt−H stretch on: (a) Pt (1 %)/Al_2_O_3_ hydrogenation catalyst, (b) Pt (10 %)/C fuel cell catalyst and (c) EuroPt1 [Pt (6 %)/SiO_2_].^18^ The latter, (c), is a difference spectrum generated by: {catalyst+H_2_}−{catalyst+D_2_}.

**Figure 5:**
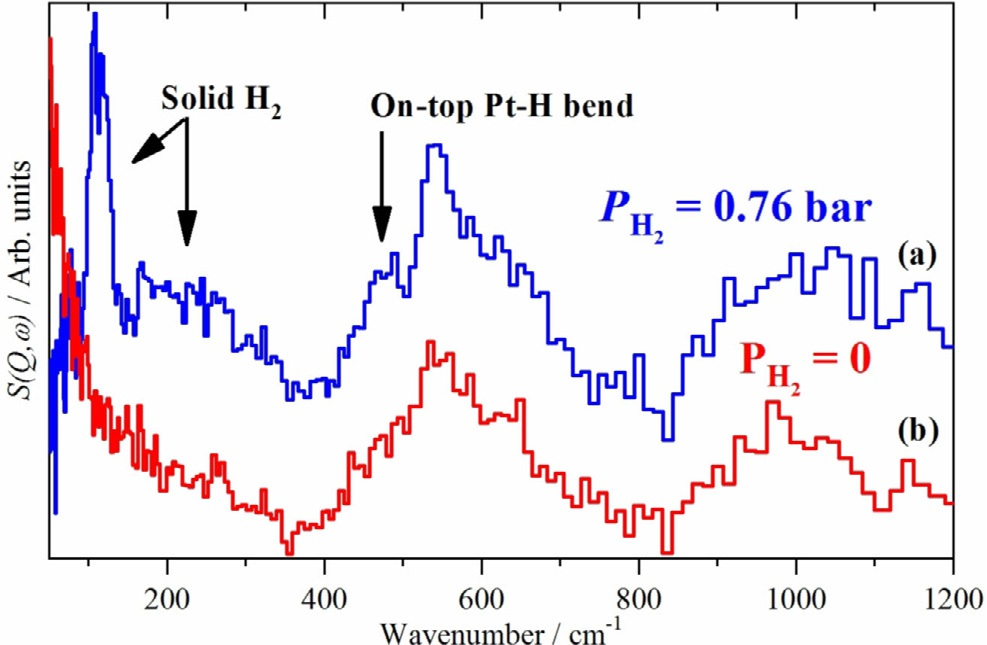
INS spectra of a Pt (58 %)/C fuel cell catalyst recorded with the TOSCA spectrometer : (a) with 0.76 bar H_2_ overpressure (b) after brief pumping at 77 K.

We note that the absence of any fourfold coordination of hydrogen is consistent with single‐crystal studies of Pt(100)8g according to which on the unreconstructed surface only twofold bridging sites are proposed. This probably because the Pt−Pt diagonal distance in the fourfold site is 3.924 Å, which is too long for the hydrogen to span. For the edge site the Pt−Pt distance is only 2.775 Å, so the hydrogen bridges the edges of the fourfold site. The twofold site has three modes: an out‐of‐plane bend, in‐plane asymmetric, and symmetric Pt‐H stretch. Inspection of the mode visualisations show that these occur in the ranges: 550–650, 900–1120, 1385–1585 cm^−1^, although there is extensive mixing with the on‐top hydrogen bending mode and the asymmetric stretch of the threefold site. (This is readily seen in Figure 3, in the 400–800 cm^−1^ region of which the same feature occurs in two or all three of the partial spectra, for example, modes at 430 and 550 cm^−1^.) The only reported values for the modes of twofold coordinated hydrogen on platinum are at 1190–1230 cm^−1[8g]^ and 950 cm^−1[11c]^ in reasonable agreement with our results.

Our result, that the INS spectrum can be assigned to a mixture of on‐top, twofold and threefold sites, accounts for all of the literature on the spectroscopy of adsorbed hydrogen on nanoparticulate platinum. Figure S1 (Supporting Information) shows a compilation from the literature and the spectra show a remarkable degree of similarity, irrespective of whether the platinum is present as high surface area metal10a–10c or as a supported catalyst.^6^, 10d, 11d,11e This strongly suggests that, in general, for adsorbed hydrogen on platinum nanoparticles, most of the hydrogen is in twofold sites. This has not been recognised previously, the spectra are generally assigned to mostly threefold hydrogen. The importance of the twofold sites is that these are proposed^20^ to be the most active sites for the hydrogen oxidation reaction.

## Experimental Section

Commercial high‐purity platinum black (98.44 %; CAS No. 7440‐06‐4) was purchased from Umicore Precious Metals Chemistry. The product specification is based on gravimetric analysis and inductively coupled plasma spectroscopy/optical emission spectral analysis (ICP‐OES). The Brunauer–Emmett–Teller (BET) surface area in is ca. 25 m^2^ g^−1^. The presence of traces of alkaline elements (Na, K) is noted in the specification. The Pt (58 %)/C catalyst was prepared by wet impregnation. High purity carbon black with a nitrogen surface area of ca. 60 m^2^ g^−1^ was used as the support. The preparation procedures for the INS measurements have been described previously.^6^ Pt (1 %)/Al_2_O_3_ and Pt (10 %)/C were purchased from Alfa Aesar. INS spectra were recorded with the TOSCA spectrometer.^21^ (ISIS, Chilton, UK) and IN1‐Lagrange (ILL, Grenoble, France).^22^ The IN1‐Lagrange data is available at: https://doi.ill.fr/10.5291/ILL‐DATA.7‐05‐441. Diffuse reflectance infrared spectra were recorded by using a Spectra‐Tech Collector with an environmental chamber fitted with KBr windows and either a Digilab FTS‐60A or a Bruker Vertex70 FTIR spectrometer. The computational studies are described in the Supporting Information.

## Conflict of interest

The authors declare no conflict of interest.

## Supporting information

As a service to our authors and readers, this journal provides supporting information supplied by the authors. Such materials are peer reviewed and may be re‐organized for online delivery, but are not copy‐edited or typeset. Technical support issues arising from supporting information (other than missing files) should be addressed to the authors.

Supplementary
