# Supplementary material for: Adsorbed States of Hydrogen on Platinum: A New Perspective
Source: Chemistry. 2019 Apr 17;25(26):6496–9. doi: 10.1002/chem.201900351 (PMC6767033; doi:10.1002/chem.201900351)
Supplement: Supplementary file 1 — Supplementary [file CHEM-25-6496-s001.pdf]

CHEMISTRY
A European Journal

Supporting Information

Adsorbed States of Hydrogen on Platinum: A New Perspective

Stewart F. Parker,*[a] Sanghamitra Mukhopadhyay,[a] Mónica Jiménez-Ruiz,[b] and
Peter W. Albers[c]

chem_201900351_sm_miscellaneous_information.pdf

1. Inelastic neutron scattering (INS) spectroscopy.

The major difference between neutron vibrational spectroscopy (inelastic neutron scattering, INS) and infrared and Raman spectroscopies is that the neutron has mass, thus an inelastic scattering event results in a significant transfer of *both* energy (ω, cm $^{-1}$) and momentum (Q, Å^{-1}). Since both ω and Q are experimentally and independently accessible, it follows that INS spectroscopy is intrinsically a two-dimensional form of spectroscopy.

INS spectroscopy can be either coherent, so gives information on collective motions of the system, or incoherent, which only involves the correlation between the position of the same nucleus at different times, hence the motions of a single particle are probed. Whether the scattering is primarily coherent or incoherent depends on the relative size of the coherent and incoherent scattering cross section's (σ) of the scattering nuclei. For incoherent scattering the INS intensity, $S(Q,\omega)$, of the *i*th vibrational mode is proportional to:[1]

$$S(Q,n\omega_i) \propto \frac{(QU_i)^{2n}}{n!} \exp\left(-(QU_{Tot})^2\right)\sigma \qquad (1)$$

where ω_i is the *i*th mode at frequency ω, n is the order of the transition, n = 1 for a fundamental, 2 for a first overtone or binary combination, 3 for a second overtone or ternary combination *etc*…, Q is the momentum transfer defined as:

$$\boldsymbol{Q} = \boldsymbol{k}_i - \boldsymbol{k}_f \text{ where } \boldsymbol{k} \equiv 2\pi/\lambda \qquad (2)$$

where the subscripts i and f refer to the incident and scattered neutrons respectively, U_i is the root mean square displacement of the atoms in the mode and σ is the inelastic scattering cross section of the atom. The exponential term in Eqn. (1) is a Debye-Waller factor, U_{Tot} is the total root mean square displacement of all the atoms in all the modes (both internal and external) and its

magnitude is in part determined by the thermal motion of the molecule. This can be reduced by cooling the sample and so INS spectra are generally best recorded below 30 K.

Equation (1) is purely mechanical; the intensity does not depend on interactions with the electrons as is the case for infrared (*via* the dipole moment derivatives) and Raman (*via* the polarisability derivatives) spectroscopies. This means that there are no selection rules and all modes are allowed. Since ^1H is the lightest isotope of the lightest element, it has the largest amplitude of motion and this combined with the fact that it has the largest incoherent scattering cross section (80.27 barn *cf* $\leq$ 5 barn for most other elements, 1 barn = 10^{-28} m^2) means that, while there are no selection rules for INS spectra, there is a strong ‘propensity rule’ such that in hydrogenous materials, modes that involve movement of hydrogen will dominate the spectrum.

The dependence of Eqn. (1) on n shows that overtone and combination modes are allowed transitions in INS spectroscopy within the harmonic approximation. This is in marked contrast to infrared and Raman spectroscopies where such modes generally only occur weakly due to anharmonicity.

2. Computational studies.

Lattice dynamics. Periodic density functional theory (DFT) calculations of the crystalline structures were carried out using the plane-wave pseudopotential method as implemented in the CASTEP code.[2,3] Exchange and correlation were approximated using the Perdew-Burke-Ernzerhof (PBE) functional, within the generalized gradient approximation (GGA). The plane-wave cutoff energy was 830 eV. As this is an isolated molecule calculation, Brillouin-zone sampling of electronic states was performed only at the Γ-point. The equilibrium structure, an essential prerequisite for lattice dynamics calculations was obtained by Broyden-Fletcher-Goldfarb-Shanno (BFGS) geometry optimization after which the residual forces were converged to zero within ±0.0047 eV $Å^{-1}$. Phonon frequencies were obtained by diagonalisation of dynamical matrices computed using density-functional perturbation theory[4] (DFPT). The INS spectra were generated from the CASTEP output using ACLIMAX.[5]

***Ab initio* molecular dynamics (AIMD).** AIMD simulations have been performed on a nanocluster consisting of 44 Pt atoms and 80 hydrogen atoms, at a single k point with periodic boundary condition and plane-wave pseudopotentials as implemented in the CASTEP code.[2,3] The ultrasoft pseudopotentials generated with the Perdew-Burke-Ernzerhof (PBE) functional within the generalized-gradient approximation (GGA) have been used. Dispersion corrections to the PBE functional (PBE+D) were included following the methodology of Tkatchenko and Scheffler.[6] A plane-wave cut off energy 950 eV with electronic energy tolerance 5.0×10^{-7} eV is used for self consistent single point electronic energy minimization. The NPT ensemble with the Nose-Hoover thermostat and Andersen-Hoover barostat were used to stabilize temperature and pressure, respectively. The Andersen method of pressure control allows the cell to change volume with temperatures by keeping the shape preserved which is suitable for nanostructure simulations. A relaxation time for the thermostat and the cell was specified as 0.5 ps and 500 ps, respectively. A MD time step of 0.5 fs was used all throughout. The temperature of the system was kept constant at 87 K.

To compare with INS spectra, the Cartesian densities of states (CDOS) has been calculated by a Fourier-cosine transformation of the time-dependent velocity autocorrelation function (VACF), $C_{j,j}$:

$$C_{j,j}(t) = (1/3)\langle \mathbf{v_j(0)} \bullet \mathbf{v_j(t)} \rangle$$

where $\mathbf{v_j(t)}$ is the velocity of the species j at time t. This calculated CDOS is then weighted by the incoherent neutron-scattering cross section of each species for comparison with the INS data. The calculated atom projected CDOS are analogous to the vibrational densities of states (VDOS) corresponding to vibrations of atoms in Cartesian coordinates rather than along bond directions, *i.e.* internal coordinates.[7] Carrying out the calculations in this way, means that the transition energies of the atomic vibrations remain similar to that obtained by diagonalising the Hessian matrix, but the information about the eigenvectors is lost due to the collective motion of all the atoms in the nanocluster. Thus intensities obtained at normal mode frequencies can be contaminated with more than one internal modes. The data analysis software MDANSE, formally called nMoldyn,[8,9] was used to calculate these CDOS.

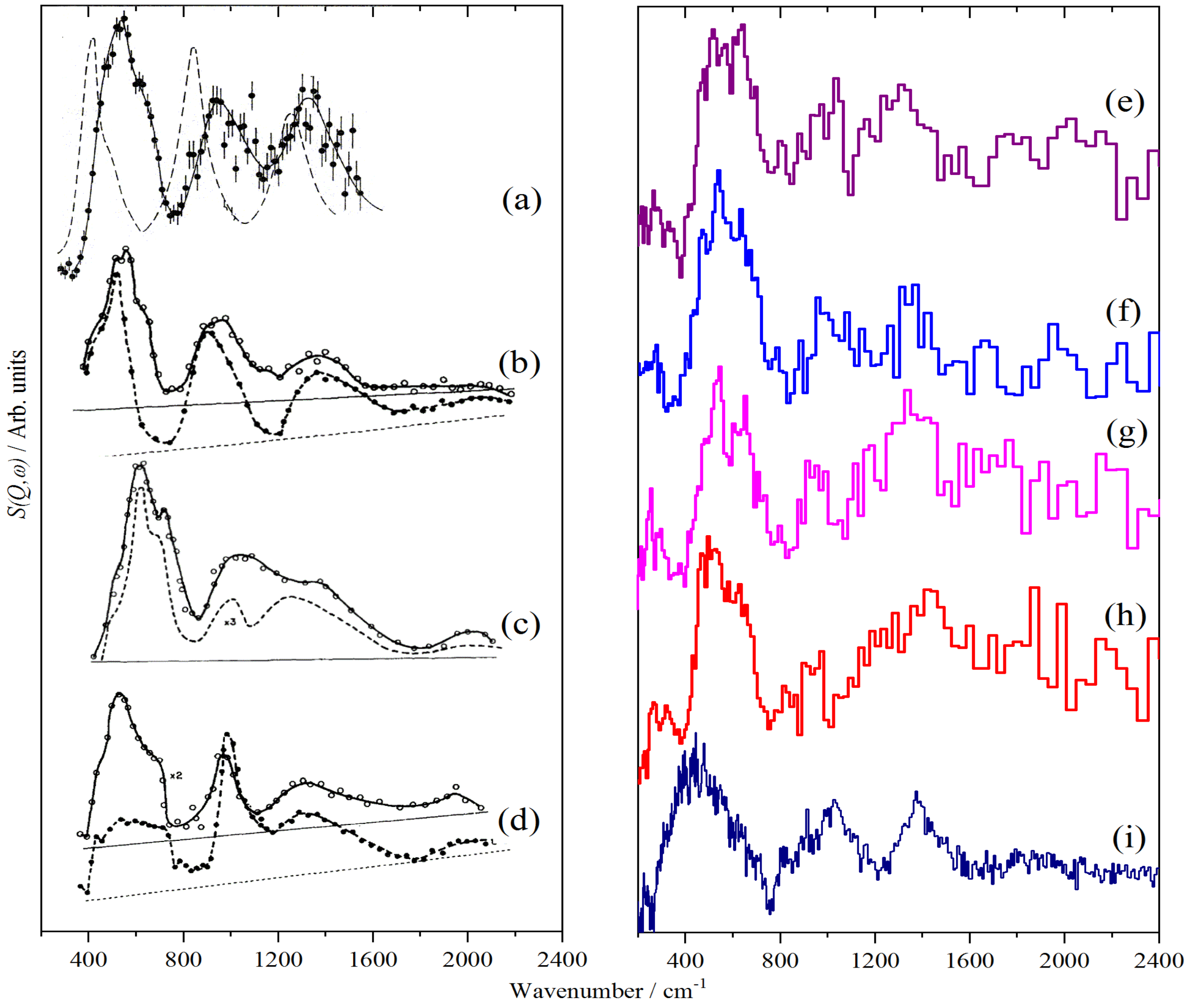

Figure S1. Compilation of INS spectra of hydrogen on platinum. (a) 1983, platinum black,[10] the dashed line is residual hydrogen on the nominally clean catalyst (BT4, NIST, USA), (b) 1988, Raney Pt,[11] the dashed line is for a coverage (θ) of 0.7 monolayers and the solid line for $\theta = 1$, (c) EuroPt1[11] (Pt(6%)/SiO_2) dashed line $\theta = 0.3$, solid line $\theta = 1$, (d) Pt(13%) in Y zeolite,[11] solid line hydrogen adsorbed at 300 K, dashed line hydrogen adsorbed at 800 K ((b) – (d) IN1-BeF, ILL, France), (e) 2006, Pt(50%)/C,[12] (f) Pt(50%)/C,[12] (g) Pt(60%)/C,[12] (h) Pt(60%)/C,[12] ((e) – (h) TOSCA, ISIS, UK, all four samples are different preparations) and (i) 2016, platinum black,(IN1-Lagrange, ILL, France). (a) is reproduced from ref. [10] with permission of the American Physical Society, (b) – (e) are reproduced from ref. [11] with permission of Elsevier and (e) – (h) are reproduced from ref. [12] with permission of Elsevier.

References

[1] P. C. H. Mitchell, S. F. Parker, A. J. Ramirez-Cuesta, J. Tomkinson, *Vibrational spectroscopy with neutrons, with applications in chemistry, biology, materials science and catalysis*, World Scientific, Singapore, **2005**.

[2] S.J. Clark, M.D. Segall, C.J. Pickard, P.J. Hasnip, M.J. Probert, K. Refson, M.C. Payne, *Z. Krist.* **2005**, *220*, 567-570.

[3] K. Refson, S.J. Clark, P.R. Tulip, *Phys. Rev. B* **2006**, *73,* 155114.

[4] V. Milman, A. Perlov, K. Refson, S.J Clark, J. Gavartin, B. Winkler, *J. Phys.: Condens. Matter* **2009**, *21*, 485404.

[5] A.J. Ramirez-Cuesta, *Comp. Phys. Comm.* **2004**, *157,* 226-238.

[6] A. Tkatchenko and M. Scheffler, *Phys. Rev. Lett.* **2009**, *102*, 073005

[7] Pin-Kuang Lai and Shiang-Tai Lin, *J. Comput. Chem.* **2015**, *36*, 507-517.

[8] https://mdanse.org/ (7th December, 2018).

[9] G. R. Kneller, V. Keiner, M. Kneller and M. Schiller, *Comput. Phys Commun.* **1995**, *91*, 191-214

[10] J.J. Rush, R. R. Cavanagh, R.D. Kelley, *J. Vac. Sci. Technol. A* **1983**, *1*, 1245-1246.

[11] A.J. Renouprez, H. Jobic, *J. Catal.* **1988**, *113*, 509-516.

[12] S.F. Parker, C.D. Frost, M. Telling, P. Albers, M. Lopez, K. Seitz, *Catalysis Today* **2006**, *114*, 418-421.
